# Adequacy of sedation analgesia to support the comfort of neonates undergoing therapeutic hypothermia and its impact on short-term neonatal outcomes

**DOI:** 10.3389/fped.2023.1057724

**Published:** 2023-03-09

**Authors:** Pauline Nakhleh-Philippe, Claire Zores, Amélie Stern-Delfils, Benoît Escande, Dominique Astruc, François Severac, Pierre Kuhn

**Affiliations:** ^1^ Department of Neonatology, University Hospital of Strasbourg, Strasbourg, France; ^2^ Department of Neonatology, Hospital of Mulhouse, Mulhouse, France; ^3^ Strasbourg University, Institut des Neurosciences Cellulaires et Intégratives, Strasbourg, France; ^4^ Department of Public Health and Epidemiology, University Hospital of Strasbourg, Strasbourg, France; ^5^ Neonatal Research Unit, Department of Women’s and Children’s Health, Karolinska Institute, Stockholm, Sweden

**Keywords:** neonate, therapeutic hypothermia, sedation, analgesia, comfort, pain, short-term outcome

## Abstract

**Objectives:**

We aimed to evaluate (1) whether sedation analgesia (SA) used during therapeutic hypothermia (TH) was efficient to support the wellbeing of neonates with hypoxic-ischemic encephalopathy, (2) the SA level and its adjustment to clinical pain scores, and (3) the impact of inadequate SA on short-term neonatal outcomes evaluated at discharge.

**Methods:**

This was an observational retrospective study performed between 2011 and 2018 in two level III centers in Alsace, France. We analyzed the wellbeing of infants by using the COMFORT-Behavior (COMFORT-B) clinical score and SA level during TH, according to which we classified infants into four groups: those with excess SA, adequate SA, lack of SA, and variability of SA. We analyzed the variations in doses of SA and their justification. We also determined the impact of inadequate SA on neonatal outcomes at discharge by multivariate analyses with multinomial regression, with adequate SA as the reference.

**Results:**

A total of 110 patients were included, 89 from Strasbourg university hospital and 21 from Mulhouse hospital. The COMFORT-B score was assessed 95.5% of the time. Lack of SA was mainly found on the first day of TH (15/110, 14%). In all, 62 of 110 (57%) infants were in excess of SA over the entire duration of TH. Most dose variations were related to clinical pain scores. Inadequate SA was associated with negative short-term consequences. Infants with excess of SA had a longer duration of mechanical ventilation [mean ratio 1.46, 95% confidence interval (CI), 1.13–1.89, *p* = 0.005] and higher incidence of abnormal neurological examination at discharge (odds ratio 2.61, 95% CI, 1.10–6.18, *p* = 0.029) than infants with adequate SA.

**Discussion:**

Adequate SA was not easy to achieve during TH. Close and regular monitoring of SA level may help achieve adequate SA. Excess of SA can be harmful for newborns with hypoxic-ischemic encephalopathy who are undergoing TH.

## 1. Introduction

Therapeutic hypothermia (TH) has now become the gold standard of care for neonates with moderate to severe hypoxic-ischemic encephalopathy (HIE). Several major cooling trials have highlighted that by allowing the recovery of the energy metabolism of the nerve cells (1), TH is an effective therapy to reduce death or disability at 18 months of age, as shown by many randomized controlled trials summarized in a systematic review (2–11). These benefits are still present in childhood at 6–7 years of life (12–14). However, TH is recognized as a significant physiologic stress in itself, as evidenced by elevated circulating cortisol and norepinephrine levels in animals (15) and discomfort and pain during TH and rewarming in humans (16). The impact of this stress and the pain may limit the neurodevelopmental benefits of TH. Indeed, in vulnerable preterm infants, neonatal pain has been implicated in the disruption of normal brain development *via* excitotoxic damage and upregulation of the hypothalamic–pituitary–adrenal axis, causing immediate and extending consequences on brain development (17) and possibly leading to motor impairment (18) and cognitive impairment (19). Moreover, in full-term infants, neonatal pain may alter pain perception toward hypersensitivity through pain memory (20, 21). The latter appears true from the first moment of life and is also affected by the mode of delivery (22). Neonatal pain management is an integral part of neonatal care since the studies by Anand et al. (23, 24). Alleviating pain is essential to not only support the wellbeing of newborns but also protect their motor, cognitive, and psychological development (25).

Providing optimal sedation analgesia (SA) while neonates are undergoing TH may be beneficial. However, the use of SA on a developing brain has recently sparked debate, particularly the neurotoxic effect of analgesics, sedatives, and other anesthetics. The use of SA in TH is not obvious, is not well codified, and is still questioned in the literature. Animal studies demonstrated controversial results. In a piglet model, moderate hypothermia decreased the severity of brain damage only if it was associated with halothane or intravenous anesthesia (26, 27). TH conducted without SA appeared to be neuroprotective in sheep (28). Wassink et al. argued that evidence was insufficient to consistently recommend the use of SA during TH in term infants (29). The adult experience of TH in cardiac arrest required SA for better performance of the technique and better maintenance of target temperatures (30–32). Adequate TH could not be achieved in adults or children without deep SA and muscle relaxation to suppress shivering. However, neonates have non-shivering thermogenesis due to excess brown fat and, therefore, sedation and muscle relaxation should not be required to induce hypothermia (33, 34). In the neo.nEURO.network randomized controlled trial, opioids (morphine 0.1 mg/kg every 4 h or equivalent dose of fentanyl) were administered to reduce discomfort attributable to encephalopathy and to counteract the stress response induced by TH, which might reduce the effectiveness of hypothermia (8). In the TOBY trial, Azzopardi et al. used analgesia with morphine or chloral hydrate on a case-by-case basis, only in the event of signs of discomfort, without describing the modalities or even the number of such events (35). In the NICHD randomized controlled trial, opioid sedation was used alone or combined with anticonvulsant drugs in 43% (*n* = 89/208) of infants with moderate or severe HIE (6). Later, the authors reported that SA induced a longer duration of ventilation and hospitalization, with no benefit on neuroprotection (36). In a recent survey of cooling centers in the United Kingdom and United States, more than 80% of centers reported preemptive opioid sedation during TH (37). Hypotension was more frequent and hospital stay more prolonged in neonates receiving than not receiving morphine. In addition, for long-term effects, morphine did not improve neuroprotection, showing a higher rate of brain damage on imaging and poorer neurodevelopmental outcome at 2 years (38). Although randomized controlled trials of TH report the benefits and limitations of the use of sedatives and analgesics in TH, they allowed SA provision at the provider's discretion. The impact of SA on the neurodevelopmental outcome after therapeutic hypothermia for HIE is unclear, and there are no established guidelines for treatment in neonates during TH.

Following the recommendations of the French Society of Neonatology, for more than 10 years, TH has been provided nationwide to all neonates over 36 weeks of gestation with HIE to improve their long-term prognosis (39). However, we lack data describing the use of SA and its concentration, increased or decreased dosage, or duration. The question of the safety of SA remains unclear. In neonatal intensive care units (NICUs) in Alsace, the protocol used by the perinatal network *Naître en Alsace* recommended the use of SA from the onset of TH.

We reviewed our SA practices to assess their efficiency and their possible impact on the newborn infants. The primary objective of this study was to evaluate the adequacy of SA of neonates undergoing TH assessed through comfort/pain scores. Secondary objectives were to assess (1) the changes in levels of SA and whether variation in SA level was adjusted or not to clinical behavioral or physiological pain indicators; (2) the potential association of inadequate SA with altered short-term outcomes of the neonates such as (i) an abnormal neurologic examination at discharge, (ii) brain lesions at MRI, (iii) a withdrawal syndrome or its prolonged treatment, (iv) an increased duration of mechanical ventilation, (v) an increased duration of hospital stay, and (vi) a delayed acquisition of feeding autonomy.

## 2. Methods

### 2.1. Participants

This study was a retrospective multicenter review of neonates admitted to NICUs at the two level III perinatal centers in Alsace between January 2011 and December 2018. Strasbourg university hospital and Mulhouse general hospital implemented similar guidelines from the perinatal network of the Alsace region. All eligible infants included in the study had been diagnosed with HIE requiring TH and received intravenous SA. Entry criteria for TH followed the French Society of Neonatology recommendations (39). The exclusion criteria were death during the first 72 h, discontinuation of TH before 72 h regardless of cause, and the continuation of SA for more than 6 days after the end of TH.

### 2.2. Sedation analgesia treatment

Since the introduction of TH in NICUs in Alsace, it was recommended in the protocol to provide SA with midazolam 100 µg/kg/h and fentanyl 1 µg/kg/h and to adapt the doses according to the COMFORT-Behavior (COMFORT-B) score. They were started at the initiation of TH as a continuous infusion. Increases in dosage and/or boluses were allowed based on the infant's comfort evaluation or the infant's medical needs.

### 2.3. Comfort and pain evaluation

To evaluate the infant's wellbeing, NICU nurses assessed neonates’ pain or distress by using the COMFORT-B Scale several times per day, usually every scheduled caregiving episode. The COMFORT-B Scale is an adaptation of the COMFORT Scale initially validated in 1992 (40) and was further developed, including only behavioral assessment. The COMFORT-B Scale includes six behavioral categories (muscle tone, facial tension, alertness, calmness/agitation, respiratory response, and physical movement). Each category uses a behaviorally anchored interval rating scale scored from 1 to 5 to produce a total score of 6–30 (41). To assess the analgesic efficacy of SA, pain was assessed by using the *Douleur Aiguë du Nouveau-né* (DAN) newborn acute pain and behavioral pain scale and the *Échelle Douleur Inconfort Nouveau-Né* (EDIN) neonatal pain and discomfort scale. The DAN scale incorporates facial expression, vocal expression, and limb movements of the newborn upon realization of a painful stimulus (42). The EDIN scale uses five behavioral indicators of prolonged pain: facial activity, body movements, quality of sleep, quality of contact with nurses, and consolability (43). Discomfort, distress, or lack of sedation was defined by a COMFORT-B score >17 or an EDIN score >5 or a DAN score >3. Excess SA was defined by a COMFORT-B score <11.

### 2.4. Ethics approval

The study was approved by the local ethical committee of the Strasbourg University Medical Faculty and the institutional review board. All parents provided written informed consent for their infants to participate in the prospective recording of medical data in the hospital's database for the unit, which was registered at the National Commission on Informatics and Liberty (CNIL) of France.

### 2.5. Data collection

General data collected included patient demographics; pertinent medication information and management for the birth; the diagnosis of HIE (including the worst Sarnat score at the onset of TH) and its therapeutic, clinical, and paraclinical neurological examinations; laboratory assessments, especially umbilical cord blood gas indices; and liver or kidney impairments. Clinical outcomes included comfort and pain scores, signs of withdrawal syndrome assessed through the Finnegan score (44), and respiratory and nutritional supports. Dosing information for midazolam and fentanyl was reported hour by hour. We also calculated the cumulative drug dose administered as continuous infusion and bolus on each day of the analyzed period. The need for supplemental analgesics or sedatives, anticonvulsant use, or other pharmacological treatment was recorded.

These data were collected on each day (D1, D2, and D3) of TH as well as at the fourth period (P4), which was considered the time of continuing SA beyond 72 h and until the SA was stopped. Additional hospital medical outcomes included neurological examination at discharge from hospital by the senior attendant neonatologist, results of the brain's MRI realized between 5 and 9 days after birth. The neonatal neurological examination was based on the assessment of the newborn's level of alertness, cranial nerve function, and motor and sensory system function and the presence of primitive reflexes.

### 2.6. Criteria for primary outcome: SA adequacy

To evaluate the SA status, we classified all neonates in the cohort into four SA groups based on the COMFORT-B score analyzed each day of SA: excess SA if the neonate presented at least a COMFORT-B score <11; lack of SA if the neonate presented at least a COMFORT-B score >17; adequate SA if COMFORT-B scores remained only between 11 and 17; and variability of SA if COMFORT-B scores showed both excess SA and lack of SA within 24 h. The patient could change groups any day. For the global analysis, a total score for all times of SA was defined and neonates were classified a second time with the following definition to evaluate the primary study outcome: final excess SA if the neonate belonged to the excess SA group for at least 2 days and was excluded from the lack of SA group any day; final adequate SA if the neonate belonged to the adequate SA group for at least 2 days; final lack of SA if the neonate belonged to the lack of SA group for at least 2 days and was excluded from the excess SA group any day; and final variability of SA if the neonate belonged to the variability of SA group for at least 2 days or belonged to the excess SA group 50% of the time and the lack of SA group the remaining 50% of the time.

### 2.7. Criteria for secondary outcomes

#### 2.7.1. Changes in levels of SA and their justification

The changes of the levels of SA were described based on their increase or decrease and their occurrence on a daily basis. Each change was evaluated in order to assess whether the variation in SA level was adjusted or not to clinical behavioral (COMFORT, DAN, or EDIN scores out of the normal range) or physiological (increased in the heart rate or in the mean arterial pressure reported on the patient's chart) pain indicators.

#### 2.7.2. Impact of inadequate SA on different neonatal outcomes

These neonatal outcomes were clinical and paraclinical neurological evaluation, withdrawal syndrome and its treatment duration, duration of mechanical ventilation, time to reach self-oral feeding, and discharge from the NICU and hospital. The neurological examination at discharge was the last report of relevant neurologic abnormalities or normal examination. These outcomes were defined as follows:

- abnormal neurological examination at discharge (yes or no);
- abnormal brain MRI based on the conclusion of the senior radiologist physician performing the MRI. This item was coded yes in case of brain lesions compatible with hypoxic-ischemic lesions such as basal ganglia and/or cortical or subcortical lesions, white matter lesions apart from hemorrhagic petechial lesions, isolated hemorrhagic petechial lesions, and brainstem or cerebellar lesions as used earlier (45), and no in their absence. No specific MRI score were used;
- presence of a withdrawal syndrome in case of at least two Finnegan scores >8 leading to a treatment by morphine (44). The duration of this treatment was also collected;
- duration of mechanical ventilation in days from the first intubation to the last extubation;
- duration of hospital stay in days from admission to hospital discharge to home; and
- time to reach full feeding autonomy defined as the time from birth to the withdrawal of a nasogastric tube with full enteral feeding.

### 2.8. Statistical analysis

Categorical data are described with number (percentage) and continuous data with mean [standard deviation (SD)] or median and interquartile range (IQR). The Gaussian character of the quantitative variables was evaluated with the Shapiro–Wilk test and graphically. Quantitative variables were compared by the Kruskal–Wallis test. Parametric chi-squared test or Fisher's exact test was used, as appropriate, to compare categorical variables. The effect of group on neurological examination outcome was assessed using a multivariate logistic regression model in order to adjust for the use of antiepileptic drugs. Results are presented as odds ratios (OR) with their 95% confidence intervals (CIs). Comparison of groups for the duration of hospitalization and duration of intubation/mechanical ventilation involved a multivariate Gamma regression model including the result of the neurological examination as an adjustment variable. Goodness of fit for the Gamma distribution was assessed with histograms and quantile plots. The results are presented as mean ratios (MRs) with their 95% CIs. *p* < 0.05 was considered statistically significant. Statistical analyses involved using R 3.5.0.

## 3. Results

### 3.1. Study population

**Figure 1:**
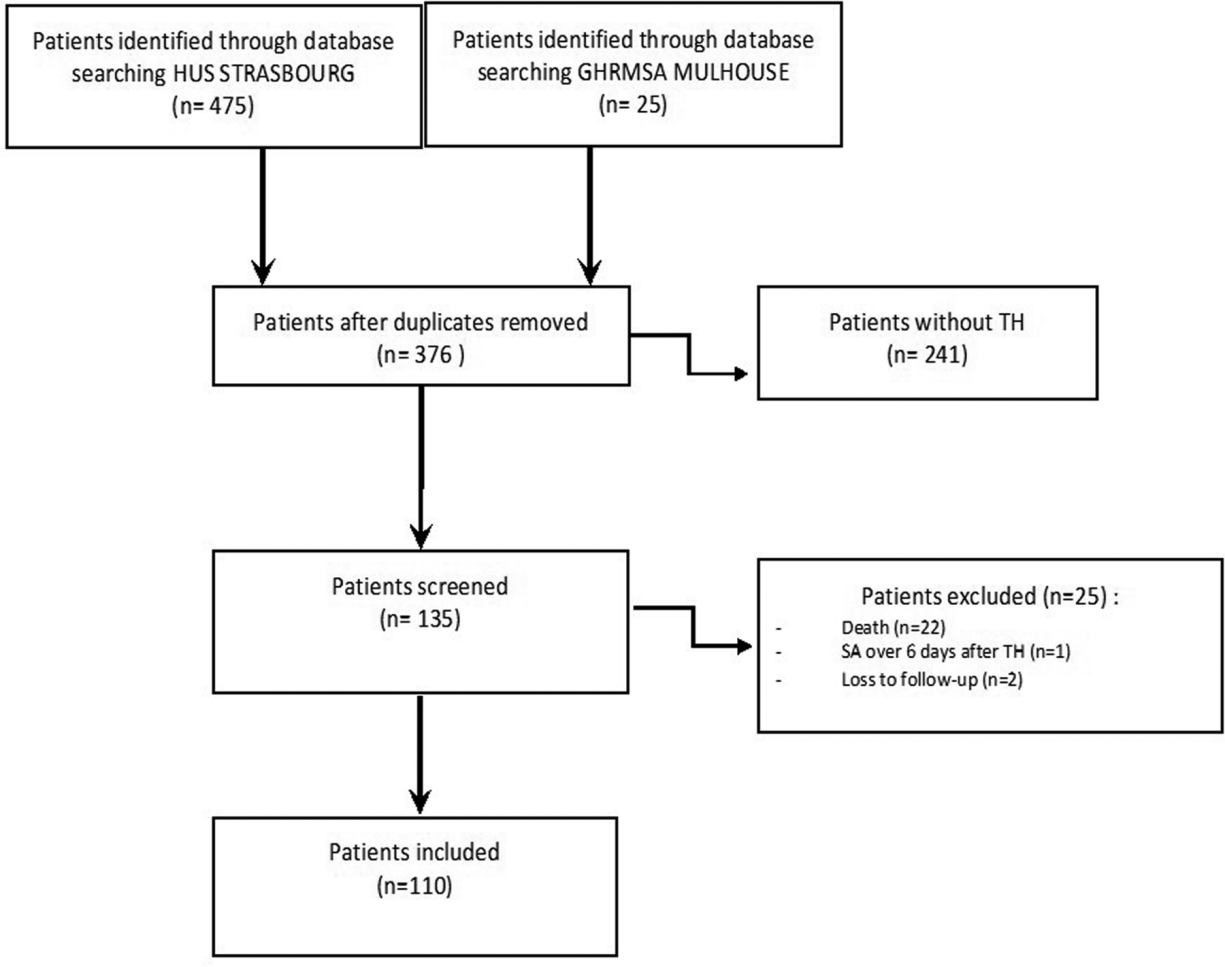
Flow of study population..

As shown in Figure 1, a total of 376 infants with HIE were identified by hospital database searching from January 1, 2011, to December 31, 2018, in Strasbourg and from January 1, 2016, to December 31, 2018, in Mulhouse. Among them, 135 were started on TH on a regular basis. We finally included 110 patients in the analysis, 89 treated at Strasbourg university hospital and 21 at Mulhouse hospital (see Figure 1 for details about exclusion). Table 1 shows the neonatal characteristics of the study population. Table 2 shows the main neonatal outcomes after TH.

**Table 1:** Study population characteristics.

Conditions of birth	
Sex ratio, male/female	54/56
Gestational age, weeks, median (IQR)	39.4 (35.5–42.2)
Birthweight, kg, median (IQR)	3.1 (1.9–4.5)
Birth by cesarean section, *n* (%)	35/110 (31.8)
Vaginal birth requiring instrumental maneuvers, *n* (%)	35/110 (31.8)
Out-born, *n* (%)	78/110 (70.8)
Apgar score at 10 min, median (IQR)	6 (0–10)
Nasotracheal intubation, *n* (%)	93/110 (84.5)
Need for epinephrine, *n* (%)	16/110 (14.6)
Cardiopulmonary resuscitation at birth, *n* (%)	41/110 (37.3)
Umbilical cord	
Arterial pH, median (IQR)	7 (6.1–7.3)
Arterial pH <7, *n* (%)	53/106 (50)
Arterial lactates, median (IQR)	10 (3.2–20)
Arterial lactates >11, number (%)	36/93 (38.7)
Neurological profile, *n* (%)	
Normal EEG voltage	15/110 (13.6)
Moderately abnormal EEG voltage	69/110 (62.7)
Severely abnormal EEG voltage	26/110 (23.6)
HIE grade 2	77/110 (70)
HIE grade 3	25/110 (13.6)
Abnormal neurological examination at discharge	55/110 (50)
Antiepileptic drugs during TH	52/110 (47.3)
Antiepileptic drugs at discharge	18/110 (16.4)
Organ failure and hemodynamics, *n* (%)	
Normal liver enzymes	20/110 (18.2)
Mild hepatic impairment (liver enzymes <3 N)	42/110 (38.2)
Severe hepatic impairment (liver enzymes >10 N)	18/110 (16.4)
Abnormal kidney failure (creatinine >90 mol/L)	46/110 (41.8)
Need for vasopressors	67/110 (60.9)
Obvious cause of pain	19/110 (17.3)

EEG, electroencephalography; HIE, hypoxic-ischemic encephalopathy; N, normal; IQR: interquartile range; TH, therapeutic hypothermia.

Moderately abnormal EEG voltage: microvoltage, discontinuous, asymmetric, and/or spikey pattern, seizures. Severely abnormal EEG voltage: very altered, suppression-burst, or almost flat pattern. Obvious cause of pain: cytosteatonecrosis, caput succedaneum, cephalohematoma, hematoma of the limbs.

**Table 2:** Main neonatal outcomes.

Neonatal outcomes Values	
Duration of the end of TH to extubation (hours:minutes), median (IQR)	56:07 (00:00–151:00)
Self-feeding duration (days), median (IQR)	3 (0–22)
Discharge from NICU (days), median (IQR)	6 (4–16)
Discharge from hospital (days), median (IQR)	12 (7–68)
Withdrawal syndrome, *n* (%)	11/110 (10%)
Duration of withdrawal treatment in days, median (IQR)	2 (0–20)

TH, therapeutic hypothermia; SA, sedation analgesia; NICU, neonatal intensive cares unit; IQR, interquartile range.

### 3.2. SA efficiency (primary outcome)

#### 3.2.1. SA treatment

The median (IQR) cumulative midazolam dose administered during TH was 2,072.2 µg/kg/day (0–6,971.1), and the median cumulative fentanyl dose administered was 20.8 µg/kg/day (0–73.3). The mean daily SA doses are presented in Table 3. Among the 110 neonates, 13 (11.8%) had an early discontinuation of midazolam infusion, before the end of TH, and 10 (9.1%) had an early discontinuation of fentanyl infusion. In the remaining neonates, the infusions were discontinued in the 24 h following the end of TH in 72 neonates for midazolam and in 70 neonates for fentanyl. Altogether, P4 lasted from a minimum of 0 h 30 min to a maximum of 132 h or 5.5 days after the end of TH for both drugs with a median or 13 h 30 for midazolam and 17 h for fentanyl. SA was continued after the end of TH for a mean of 18 h 38 min ± 22 h 22 min for midazolam and 21 h 45 min ±22 h 35 min for fentanyl. Other neonatal outcomes after SA are shown in Table 3.

**Table 3:** SA doses used.

	D1	D2	D3	P4
**Midazolam**
Mean dose (µg/kg/h)	86.5 ± 31	94.4 ± 46	81.3 ± 45	—
Minimum dose (µg/kg/h)	64.4 ± 27.6	78.4 ± 39.6	62.6 ± 39.5	26 ± 19.9
Maximum dose (µg/kg/h)	103.7 ± 37.6	100.2 ± 46.5	91.4 ± 49.3	60.8
Cumulative dose (µg/kg/day)	2,075.5 ± 743.1	2,266.1 ± 1,105.7	1,951.7 ± 1,079.7	1,102.6 ± 1,776.6
**Fentanyl**
Mean dose (µg/kg/h)	0.9 ± 0.3	1 ± 0.3	0.9 ± 0.3	—
Minimum dose (µg/kg/h)	0.8 ± 0.5	0.8 ± 0.4	0.7 ± 0.4	0.4 ± 1
Maximum dose (µg/kg/h)	1.1 ± 0.7	1.0 ± 0.5	0.9 ± 0.5	0.9 ± 1.9
Cumulative dose (µg/kg/day)	21.7 ± 7.7	23.2 ± 11.6	20.2 ± 11.6	13.6 ± 21.4

SA, sedation analgesia; TH, therapeutic hypothermia; D1, D2, D3, days 1, 2 and 3 of TH; P4, period 4 from the end of TH to stopping SA; SD, standard deviation; midazolam and fentanyl doses in µg/kg/h.

Data are mean ± SD.

#### 3.2.2. Analysis of clinical pain scores

**Figure 2:**
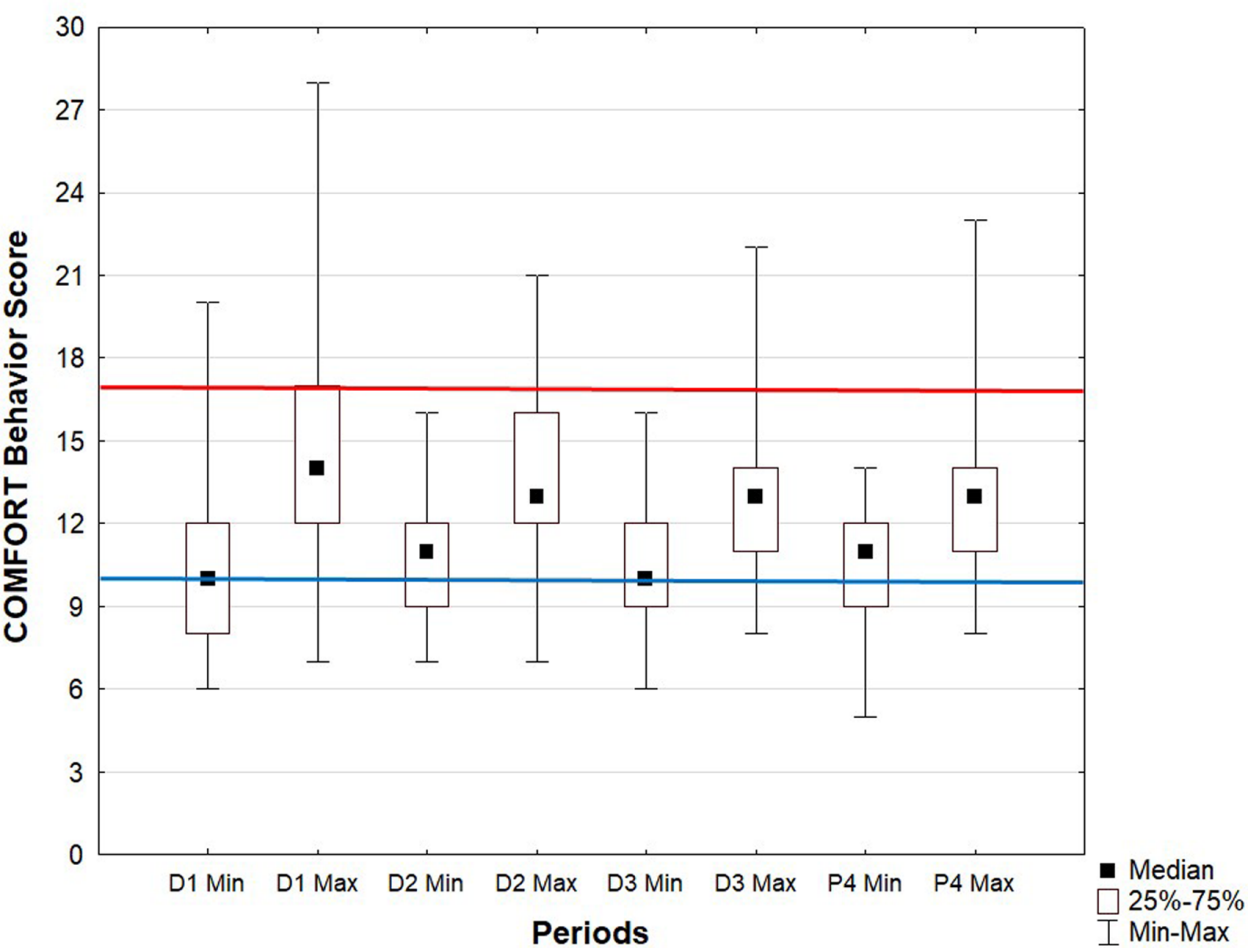
COMFORT-Behavior Scale score every day of therapeutic hypothermia. Data are median, interquartile range and minimum and maximum values. SA, sedation analgesia.

The wellbeing of neonates was assessed by the COMFORT-B Scale in 100% of neonates at the initiation of treatment and more than 96% in the following days. Pain and discomfort were also analyzed by the EDIN and DAN scores. The EDIN score was assessed in 40% of patients at the initiation of treatment and in 40.9% at the end of treatment, and in less than 20% on the other 2 days. The DAN score was assessed in less than 15% of patients. The COMFORT-B score was assessed from 0 to 6 times a day (median of four times a day). Across all 4 days, the mean COMFORT-B score was close to excess sedation: 11.9 ± 0.5, from 11.4 ± 1.8 at D3 to 12.3 ± 2.7 at D1. The mean minimal COMFORT-B score was 10.4 ± 0.3, from 10 ± 2.1 at D3 to 10.6 ± 2 at D2, and mean maximal COMFORT-B score was in the target range of the COMFORT-B score: 13.6 ± 1, from 12.7 ± 2.4 at D2 to 14.9 ± 4 at D1. Figure 2 shows the medians of these scores between D1 and P4 and the minimum and maximum values on each day.

#### 3.2.3. Distribution of neonates by SA adequacy group

**Figure 3:**
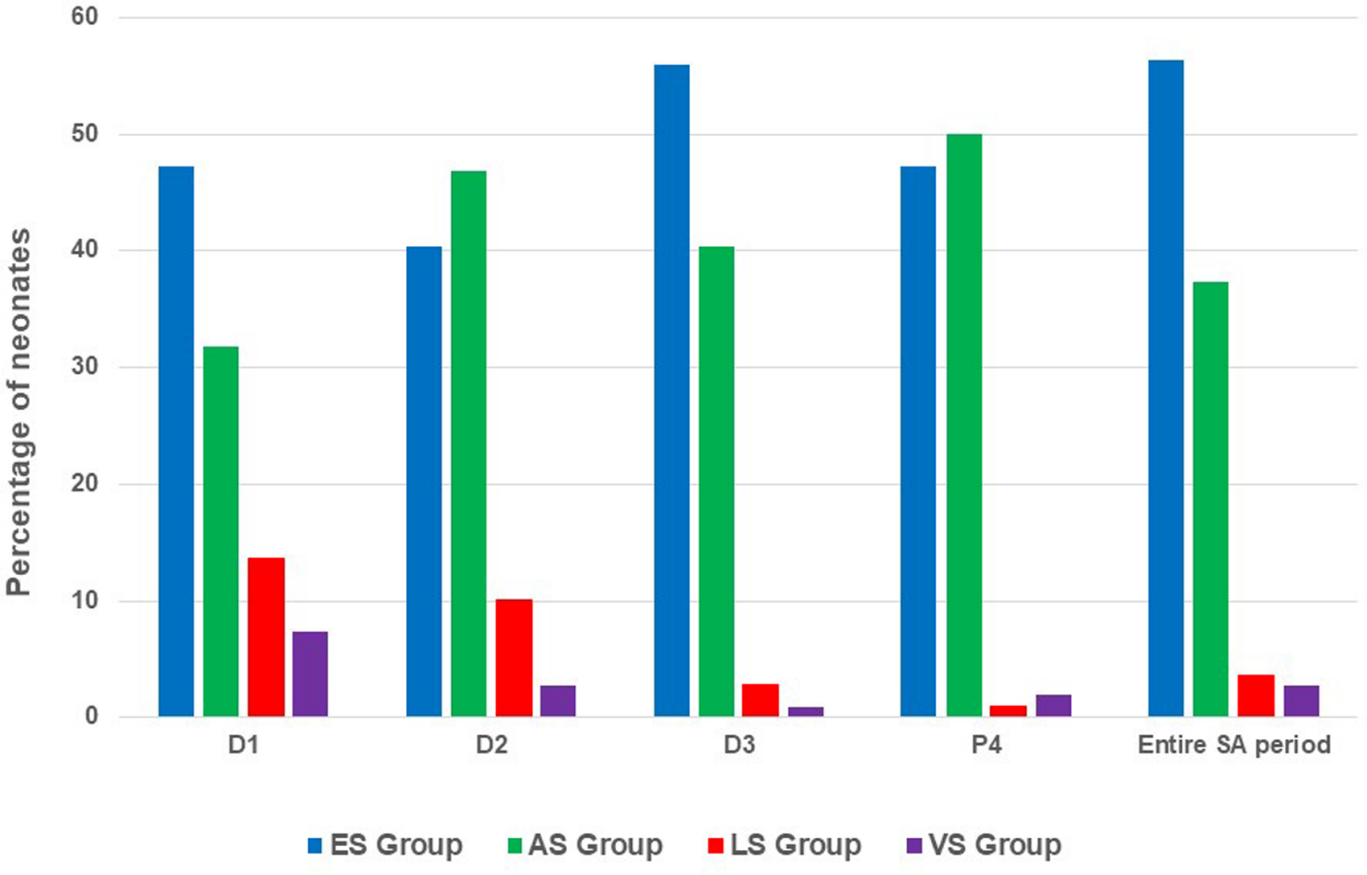
Distribution of wellbeing of neonates. ES, excess SA; AS, adequate SA; LS, lack of SA; VS, variability of SA; SA, sedation analgesia.

The distribution of the infants in the four groups of SA adequacy based on COMFORT-B scores are presented in Figure 3. A total of 62/110 (56.6%) infants were classified in the final excess SA group over the entire period of SA and 41/110 (37.3%) had a COMFORT-B score within the normal target range (i.e., wellbeing without excess or deficiency of SA; final adequate SA group) over the study period. Only 4/110 (3.6%) infants belonged to the final lack of SA group. The lack of SA group was more represented at D1 (*n* = 15/110, 13.6%). Neonates with variable SA status over 24 h, who could have a score <11 as well as a score >17 over 24 h, were poorly represented: scores for the final variability of SA group (*n* = 3/110, 2.6%) ranged from 7.3% (*n* = 8/110) on D1 to 0.9% (*n* = 1/110) on D3. Overall, 75% of infants changed groups at least once. Among the final excess SA group, 27.4% (*n* = 17/62) were always over-sedated, 35.5% (*n* = 22/62) were over-sedated half of the time, and 37.1% (*n* = 23/62) were over-sedated three quarters of the time. Among the final lack of SA group, all neonates were in lack of SA for at least 2 days.

### 3.3. Secondary outcomes

#### 3.3.1. Agreement between variations in SA level and clinical pain indicators

The increase in SA doses was mainly on D1 (*n* = 77/110, 70%), then on D2 (*n* = 50/110, 45.5%), with an increment reduction on the following days (Table 4). After the end of TH, the dosing increase concerned 6.4% (*n* = 7/110) of neonates. Continuous dose escalation was associated with boluses (i.e., discontinuous dose escalation). The boluses were mainly given on D1 in half of the population, from 47.3% (*n* = 52/110) for midazolam to 49.1% (*n* = 54/110) for fentanyl. The distribution of boluses progressively decreased every day, to 8.2% (*n* = 9/110) for midazolam and 9.1% (*n* = 10/110) for fentanyl.

**Table 4:** Sedation analgesia management assessed through the number (percentage) of neonates affected by dose variations.

	Day 1	Day 2	Day 3	Period 4
Increasing dose	77 (70)	51 (46.4)	19 (17.3)	7 (6.4)
Decreasing dose	63 (57.27)	50 (45.5)	80 (72.7)	90 (81.8)
Bolus of midazolam	54 (49.1)	37 (33.6)	33 (30)	9 (8.2)
Bolus Of fentanyl	53 (48.2)	43 (39.1)	38 (34.5)	10 (9.1)

Data are n (%).

The dosing increase was justified in 33.5% of cases by inadequate clinical scores. Hemodynamic criteria (increase in heart rate or mean arterial pressure) justified 31.4% of the increases. Almost one quarter (21.5%) of the dosing increases were unjustified by physiological or behavioral pain indicators over the entire study period.

SA doses were decreased throughout the TH treatment period, mainly on the last 2 days (72.2% of neonates on D3, 81.8% at P4). On D1, more than half of the neonates (57.3%) had at least a reduction of drug doses. The decrease in SA doses was mainly justified by inadequate clinical scores (48.5% over the whole study period), essentially on the first 3 days. At P4, dose reductions were not justified in 46.2% of cases.

#### 3.3.2. Association of inadequate SA with short-term neonatal outcomes

Using a univariate analysis, the four groups did not differ on their baseline characteristics, particularly in neurological degree of HIE defined by the highest Sarnat score (45) before TH was started (*p* = 0.205) and the first electroencephalography (EEG) assessment (*p* = 0.101). Moreover, the additional administration of antiepileptic drugs did not significantly differ between groups: final adequate SA, 15/41 (36.6%); final excess SA, 35/62 (56.5%); final lack of SA, 1/4 (25%); and final variability of SA, 1/3 (33.3%), *p* = 0.154.

When considering the potential association of inadequate SA with altered short-term neonatal outcomes, the four groups differed in an abnormal neurological examination at discharge: final excess SA, 38/62 (61.3%); final adequate SA, 14/41 (34.1%); final lack of SA, 2/4 (50%); and final variability of SA 1/3 (33.3%), *p* = 0.026. MRI was not a distinguishing feature among groups (*p* > 0.05), nor was withdrawal syndrome or duration of its treatment (all, *p* > 0.05). The duration of mechanical ventilation differed but not significantly among groups (*p* = 0.07). SA status had an impact on the duration of hospital stay (*p* = 0.04) unrelated to discharge from the NICU (*p* > 0.05). We observed also differences in time to both food and respiratory autonomy (*p* > 0.05). (Table 5) shows the distribution of outcomes significantly affected by SA status during the entire period of SA.

**Table 5:** Multivariate analysis of outcomes significantly related to sedation analgesia status during the entire period of SA.

	Final excess SA group	Final adequate SA group	Final lack of SA group	Final variability of SA group	*p*-value
	(*n* = 62)	(*n* = 41)	(*n* = 4)	(*n* = 3)	
Abnormal neurological examination at discharge, *n* (%)	38 (61.29)	14 (34.15)	2 (50)	1 (33.33)	0.03
Discharge from hospital (days)	16.69	13.02	18	9.67	0.04
Duration of mechanical ventilation (hours:minutes)	48:29	30:46	42:18	33:08	0.07

SA, sedation analgesia.

On multivariate analysis and as compared with adequate SA status, final excess SA status was associated with abnormal neurological examination at discharge (OR 2.61, 95% CI, 1.10–6.18, *p* = 0.029) and longer mechanical ventilation (MR 1.46, 95% CI, 1.13–1.89, *p* = 0.005) but not with longer duration of hospital stay (MR 1.19, 95% CI, 0.97–1.47, *p* = 0.096). Final lack of SA or final variability of SA status did not change the findings for neurological examination at discharge (OR 2.39, 95% CI, 0.28–20.39, *p* = 0.427; MR 1.01, 0.08–13.46, *p* = 0.997) or duration of mechanical ventilation (OR 1.42, 95% CI, 0.75–2.71, *p* = 0.287, MR 1.07, 0.51–2.24, *p* = 0.849).

Finally, the additional use of antiepileptic drugs was associated with abnormal neurological examination at discharge (OR 3.52, 95% CI, 1.55–7.98, *p* = 0.003). The more pathological the neurological examination, the more prolonged the ventilation (MR 1.49, 95% CI, 1.17–1.90, *p* = 0.002) and the more delayed the discharge from hospital (MR 1.35, 95% CI, 1.11–1.64, *p* = 0.003).

## 4. Discussion

Our study provides a systematic evaluation of the management of SA of neonates undergoing TH in two level III neonatal centers using the same SA protocol. This analysis of the pain/comfort based on the COMFORT-B score showed that a few neonates had pain scores consistent with a painful experience. The values of this score fluctuated mainly between excess SA and adequate SA from the initiation of the TH to the end of the SA. Dose variations were mostly justified by clinical pain scores (COMFORT-B, EDIN, and DAN), despite persistent dosage adjustments without identified reasons in almost one quarter of infants. Inadequate SA status during TH seemed to be associated with altered short-term neonatal outcomes. Infants with excess SA required longer mechanical ventilation and exhibited a higher incidence of abnormal neurological examination at discharge than other infants, although the use of antiepileptic drugs was also associated with abnormal neurological outcome and could have affected the excess SA.

This study had some limitations. First, because of the retrospective study design, some data were missing. Because of the lack of specific pain scores (EDIN and DAN), interpretation of the SA status mainly based on the COMFORT-B score is difficult, and the discomfort of infants could be underestimated. Second, our analysis of SA status was based on a clinical score, with only the observation of caregivers. However, its scientific validity allowed us to interpret it accurately. Finally, the inhomogeneous distribution of the number of infants in the four groups of SA status limited our analysis, although our cohort was large and exhaustive.

The study has many strengths. It addresses a question of particular importance for clinicians dealing with the management of SA during TH. Our cohort was multicentric, from two III level centers, with similar practices, which allowed for a homogeneous assessment of the concerned population benefiting from uniform management of TH. The data collection was meticulous according to a systematic analysis of the care diagram and the medical prescription, hour by hour, covering the entire TH duration. Altogether, these elements allowed us to further discuss our findings.

National guidelines for managing SA during TH are lacking. Presumed loss of neuroprotection without sedation was the most common reason clinicians gave for initiating preemptive SA (37). In fact, MRI studies of neonates receiving opioids revealed significantly less brain damage in all regions studied (46), and better long-term neurologic outcomes were described without significant long-term detrimental effects.

Regarding the choice of sedative molecules, most studies favored the use of morphine (47), either systematically (8) or on a case-by-case basis (7). However, fentanyl was also the first drug of choice (85.7%) in a national survey from Italy, followed by midazolam alone or combined with an opioid for pain management during TH in newborns (48). Fentanyl use is common in NICUs because it is associated with less sedative or hypotensive effects and has reduced effects on gastrointestinal dysmotility or urinary retention as compared with morphine, although it could be related more to tolerance disorders and increased risk of withdrawal syndrome from opioids (49). Analgesia with fentanyl, used in this study, which acts on the mu receptor, is 50–100 times more potent than morphine. The use of paracetamol as a co-analgesic of opioids could significantly decrease the need for opioids with no difference in the clinical score in premature infants (50). However, the hepatic metabolism of paracetamol could be a reason for its limited use in newborns undergoing TH, especially in case of a high proportion of hepatic impairment as observed in our study. To our knowledge, no study has analyzed in detail midazolam dosages and its use during TH. Yet, the use of midazolam is common in European NICUs (51) although questions were raised about its safety. A recent study showed improved survival with continuous midazolam infusions associated with opioids in very premature infants during initial mechanical ventilation that continued past 24 h of life, with no difference in moderate or severe sensorimotor impairments at age 2 years (53). Some recent analyses reported the potential benefits of a highly selective *α*2-adrenoreceptor agonist, dexmedetomidine, for its neuroprotective, analgesic, anti-inflammatory, and sympatholytic properties (53–56). At low dose, dexmedetomidine may present an appealing alternative sedation option in patients requiring TH and without acute adverse events (53) and even has benefits such as earlier extubation (56). However, such promising data should be verified in larger populations. Randomized controlled studies are under way (55). Other alternatives such as clonidine might be beneficial (57). Recent new proposals for guidelines stress also the fundamental importance of non-pharmacologic strategies, the avoidance of benzodiazepine, and a reduced use of opioids, potentially with the alternative dexmedetomidine (58).

Our findings indicate that the SA doses were in accordance with the recommended doses of the protocol. This contrasts with data in the literature reporting wide disparities in the use of SA. Differences went up to 100 times the initial, daily, cumulative, and maximum opioid infusion dosage in mechanically ventilated neonates (59). This observation was confirmed in a more recent study with the largest number of neonates under TH (47). In our study, bolus SA seemed to be an alternative to an increase in continuous-flow SA. A study comparing continuous infusions of fentanyl to intermittent boluses showed that continuous infusion produced steady serum concentrations, whereas intermittent boluses produced wide fluctuations in serum concentration with high-peak concentrations in term newborns (60).

Regardless, SA must be used with caution because high and potentially toxic serum SA concentrations were described in neonates undergoing TH. Even if morphine was used at common infusion rates, its clearance has been found altered in TH. Hepatic and renal hypoxic injury after birth asphyxia provides additional impediments to drug clearance (61–63).

The SA therapeutics used for TH vary considerably, and the effects of SA on long-term neurodevelopment remain controversial with some studies being worrying (36, 38) and others being reassuring (64). Analgesic treatment seems legitimate because newborn infants could experience pain, as illustrated in this study, despite SA. Parents also support this approach, and more than one-third of the parents felt that TH was uncomfortable for their infant (65). A conservative use to achieve the desired SA level seems reasonable.

On the basis of the COMFORT-B score, the current protocol did not meet the goal of no pain. Neonates were rarely but not never painful. The lack of SA was most often found on the first day of life at TH onset. This observation calls for particular vigilance when initiating TH. A period of adaptation by the newborn to the hypothermic environment seems necessary. In addition, important interindividual differences in metabolism or discomfort related to neurological damage render dosage recommendations difficult but could explain a necessary period of adjustment to find the optimal SA dose. If on D2, 10% of the patients still lacked SA, the rate was lower than 3% in the following days. The pain seemed better controlled over time. Above all, over the entire study period, more than half of the infants exhibited excess SA. Excess SA on D1 could be explained by an initial dosage that was too high or by prior SA not considered in the study, such as premedication for intubation or SA started before neonatal transport for out-born babies. Intermittent bolus sedation could also be implicated because it was applied to almost half of the population at the initiation of TH. Not surprisingly, the administration of antiepileptic drugs played a role in excessive sedation. However, the occurrence of neonatal seizure may be associated with a more severe encephalopathy with a higher Sarnat score, which could lead to lower COMFORT-B scores in neonates seeming therefore to have excessive SA. Nevertheless, treatment of seizures during TH has been shown to improve long-term neurodevelopmental outcomes, regardless of the HIE severity (66). This strategy still remains despite a recent Cochrane review suggesting that current trials of neonatal seizure treatment options were not sufficiently sized or powered to detect clinically important reductions in mortality and severe neurodevelopmental disabilities (67).

Neonates receiving antiepileptic drugs exhibiting more excess SA may be explained by the sedative properties of this class of drugs or also by the pharmacokinetics of these therapeutics. The clinical neurological assessment performed as early as possible could be biased by excess SA administration leading to a wrongly judged abnormal neurological examination and a longer hospital stay, which may cloud the overall clinical picture and the prognosis (68). Most neonate scores were in the COMFORT-B target range on D2 and P4. However, we found very high variation in SA status for the same individual over the four periods, which reflects the difficulty of finding the optimal dose. However, SA level decreased throughout the protocol, which testifies to a desire not to over-sedate neonates. The dose variations mainly based on clinical scores were still unjustified, especially after the treatment was stopped.

Our data support a potential alteration of the short-term outcomes of newborns associated with excess SA. Our results agree with data reported by Natarajan et al. on longer duration of ventilation and hospitalization induced by SA-based opioid sedation alone or combined with anticonvulsant drugs (36). Abnormal neurological examination at discharge but not abnormal MRI findings was associated with excess SA during the entire duration of TH alone but also with the additional use of antiepileptic drugs. As already discussed, the possible impact of excess SA during TH on the neurodevelopment of newborns with HIE warrants further evaluations.

Pain management could be optimized by a continuous and monitored evaluation. The ideal clinical score for SA management in TH is not yet known. Diversifying the scores for a more reproducible and objective evaluation could be a solution. The Neonatal Facial Coding System (NFCS) or the Neonatal Pain, Agitation Sedation Scale (N-PASS) tool seem adapted to the silent or sleeping child (69). Further studies should specifically evaluate the effectiveness and usefulness of these measures in TH. A multimodal approach would further support understanding the pain newborns experience during TH. Because the perception of the caregiver is crucial and irreplaceable, a better assessment will probably allow for a faster, more efficient, and more adapted management of SA. This could be done autonomously by the caregiver in charge of the child, following a decision chart allowing for better management of pain, by a morphine pump mediated by the nurse, on the first day of TH. Such an algorithm to adjust SA levels systematically could be used for future improvements. Indeed, Nurse- (or parent-) controlled analgesia in newborn infants has shown some potential benefits and may reduce the amount of opioid analgesia required without compromising pain relief or increasing the risk of adverse events (70). More research is needed to further evaluate this strategy. Moreover, better knowledge about the specific pharmacokinetics of sedative and analgesic drugs are expected in order to better titrate SA. Finally, the development of a pain management protocol specifically for TH may be associated with less variation in median daily opioid doses and less opioid dose escalation in ventilated infants, as has been shown in other studies (71).

## 5. Conclusion

Our study indicated that the adequacy of SA during TH could be improved because scores for only a few infants were in the normal expected range of comfort during the whole period of TH. Despite adjustments of SA level, many infants continued to exhibit excess SA. This excess SA was associated with some poor neonatal clinical outcomes. However, the use of SA appears legitimate. Recent reassuring data have shown that the use of morphine and fentanyl during TH did not impair the neurodevelopment of newborn infants (64). The evaluation of pain and support of the wellbeing of neonates have become an integral part of NICU management. The alterations in consciousness by potential lesions due to HIE and by poorly balanced SA level challenged our practices. The optimal approach to ensuring the most adjusted SA level deserves particular attention. Various pharmacologic but also non-pharmacologic strategies could help achieve adequate SA. Future studies are warranted to evaluate the benefit of their implementation in the specific context of TH (58).

## Data Availability

The raw data supporting the conclusions of this article will be made available by the authors, without undue reservation.
